# GelMA‐based 3D spheroids recapitulate transcriptomic and functional hallmarks of myeloid sarcoma

**DOI:** 10.1002/2211-5463.70321

**Published:** 2026-08-13

**Authors:** Nicolas Germain, Elise Buret, Le Hoang Thanh Nguyen, Marion Cocheteux, Nicolas Touya, Mélanie Dhayer, Romain Dubois, Frank Lafont, Sebastien Janel, Patrice Maboudou, Steve Lancel, Philippe Marchetti

**Affiliations:** ^1^ University of Lille, CNRS, Inserm, CHU Lille, Institut de Recherche Contre le Cancer de Lille, UMR9020 – UMR‐S 1277 – Canther Cancer Heterogeneity, Plasticity and Therapy Resistance France; ^2^ Banque de Tissus, Centre de Biologie‐Pathologie CHU Lille France; ^3^ Institut de Pahtologie, Centre de Biologie‐Pathologie CHU Lille France; ^4^ University of Lille, CNRS, Inserm, CHU Lille, Institut Pasteur de Lille, U1019 – UMR 9017 – CIIL – Center for Infection and Immunity of Lille France; ^5^ Service de Biochimie, Centre de Biologie‐Pathologie CHU Lille France; ^6^ University of Lille, Inserm, CHU Lille, Institut Pasteur de Lille U1167‐RID‐AGE‐Facteurs de Risque et Déterminants Moléculaires des Maladies Liées au Vieillissement France

**Keywords:** bioink, bioprinting, chloroma, extramedullary myeloid leukemia, granulocytic sarcoma

## Abstract

Myeloid sarcoma (MS) is a rare extramedullary manifestation of acute myeloid leukemia (AML). Conventional two‐dimensional cultures fail to replicate the constraints of the MS microenvironment. We demonstrated that 5% gelatin methacrylate (GelMA) hydrogels provide a tunable three‐dimensional matrix for *in vitro* modeling of MS. GelMA hydrogels exhibited tissue‐like stiffness and oxygen diffusion properties, supporting the formation of viable spheroids from myeloid leukemia cell lines. The 3D context induced G1 cell cycle arrest and apoptotic progression, reflecting dormancy at extramedullary sites. RNA sequencing revealed transcriptional reprogramming in GelMA‐embedded spheroids, with an enrichment in ECM remodeling and metabolic pathways. Furthermore, transcriptomic profiles from 3D‐cultured cells aligned with patient‐derived MS samples, validating the model's relevance to MS. Thus, we present a GelMA‐based system which provides a clinically relevant platform for investigating MS biology and screening potential therapeutic strategies.

## Graphical abstract

GelMA 5% hydrogels support the formation of myeloid leukemia spheroids that recapitulate MS‐specific features, including G1 arrest, apoptosis, and ECM‐driven transcriptomic reprogramming. The 3D model mimicked soft‐tissue‐like stiffness and oxygen conditions, and transcriptomic convergence with primary MS samples confirmed its utility as a preclinical platform for studying extramedullary leukemogenesis.

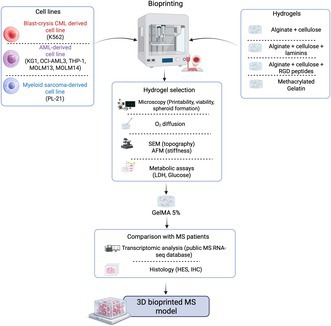

**Abbreviations**
2D: two‐dimensional
3D: three‐dimensional
AFM: atomic force microscopy
AML: acute myeloid leukemia
DPBS: Dulbecco's phosphate‐buffered saline
ECM: extracellular matrix
EdU: 5‐ethynyl‐2′‐deoxyuridine
EM: extramedullary
FBS: fetal bovine serum
FDG PET/CT: fluorodeoxyglucose–positron emission tomography/computed tomography
GelMA: gelatin methacrylate
GO: gene ontology
GSEA: gene set enrichment analysis
LAP: lithium phenyl‐2,4,6‐trimethylbenzoylphosphinate
LDH: lactate dehydrogenase
MMP: matrix metalloproteinase
MRI: magnetic resonance imaging
MS: myeloid sarcoma
NES: normalized enrichment score
NGS: next‐generation sequencing
PCA: principal component analysis
PFA: paraformaldehyde
PI: propidium iodide
RGD: Arginine‐glycine‐aspartic acid
RNA‐seq: RNA sequencing
SEM: scanning electron microscopy

Myeloid sarcoma (MS), also known as chloroma, is a rare extramedullary acute myeloid tumor characterized by immature myeloid cells that form tumor masses in extramedullary (EM) tissues. MS commonly involves soft tissues, bones, lymph nodes, and the gastrointestinal tract, with less frequent occurrence in the genitourinary system, central nervous system, and skin [1]. The clinical presentation varies based on tumor location and size, often mimicking solid tumors or lymphomas. MS may occur in isolation before the development of systemic leukemia or intramedullary acute myeloid leukemia (AML). Most newly diagnosed MS cases eventually show bone marrow involvement, whereas others arise from pre‐existing myelodysplastic syndromes, myeloproliferative neoplasms, or chronic myelomonocytic leukemia [2, 3, 4]. Diagnosing MS is challenging because of its variable presentation and the frequent absence of bone marrow involvement. Isolated MS may mimic lymphoma or tumors, leading to misdiagnosis in half of the cases [4]. Diagnosis requires histological and immunophenotypic confirmation through molecular testing. Histologically, MS consists of poorly differentiated myeloid blasts with monoblastic phenotypes that form diffuse sheets of atypical cells with a high nuclear‐to‐cytoplasmic ratio and prominent nucleoli [5, 6]. Immunohistochemical analysis is crucial for diagnosis, with MS showing myeloid lineage markers, such as myeloperoxidase and lysozyme, distinguishing it from other cell neoplasms. Molecularly, MS shows targetable mutations, with NGS revealing alterations in NPM1, NRAS, DNMT3A, IDH1/2, and FLT3 and notably higher IDH1 mutations in EM samples than in bone marrow (26% vs. 3%), highlighting the importance of EM‐site genomic profiling for targeted therapy [7]. FDG‐PET/CT and MRI can aid in the detection and monitoring of lesions. MS is difficult to treat because it combines solid tumor behavior, hematological malignancy features, and microenvironmental protection mechanisms. These factors make it a sanctuary site for relapse, leading to poor outcomes even with aggressive therapies. Systemic AML‐like chemotherapy remains the primary treatment, even for isolated MS, as local therapy yields poor outcomes [8].

Modeling myeloid sarcoma is essential for understanding its biology, identifying therapeutic targets, and developing effective treatments. However, the lack of representative models, the complexity of extramedullary niches, and the rarity of the disease pose major obstacles. Few cell lines reliably reproduce the features of MS in culture. Most preclinical AML models do not develop solid tumors, making MS‐specific studies difficult. Given the complex architecture of MS, conventional 2D culture systems cannot replicate its spatial and microenvironmental features. Standard 2D cultures lack essential factors such as extracellular matrix stiffness, oxygen gradients, and cell–cell or cell‐stromal interactions. Advanced models (e.g., 3D bioprinting and organ‐on‐chip) are still rare and underdeveloped in MS. 3D culture methods, including spheroids and organoids, better replicate tumor tissue architecture within a matrix‐mimicking extracellular matrix. These systems provide an accurate representation of cell interactions and spatial organization, with multicellular spheroids emerging as a promising approach for studying tumor biology *in vitro*. Spheroids recapitulate key features of the tumor microenvironment, including hypoxic gradients, nutrient diffusion barriers, and stromal architecture, which influence tumor behavior and therapeutic responses [9]. Spheroids reflect tumor complexity more accurately than 2D monolayers, making them relevant for studying cancer pathophysiology and resistance mechanisms [10]. They serve as platforms for drug screening and the preservation of cancer stem cell populations, bridging traditional cell lines and patient‐derived xenografts [11]. ECM‐mimicking hydrogels promote spheroid formation *in vitro* by providing structural support and biochemical cues that regulate tumor cell behavior. Although natural matrices such as Matrigel are widely used, their undefined composition and batch variability limit reproducibility [12]. Synthetic and semi‐synthetic hydrogels with tunable properties have been developed, and 3D bioprinting platforms enable the spatial control of cell placement in tissue‐like constructs [13, 14]. Gelatin Methacrylate (GelMA) retains RGD cell‐adhesive motifs and MMP‐sensitive sequences for cell–matrix interactions [15, 16]. At a concentration of 5%, it exhibited bone marrow‐like stiffness (~ 1.5 kPa), which is suitable for modeling hematological malignancies. With the LAP photoinitiator, GelMA enables visible‐light polymerization while maintaining cell viability, making it effective for studying diseases such as myeloid sarcoma [17].

In this study, we systematically compared a panel of natural and semi‐synthetic hydrogels to identify the most suitable matrix for *in vitro* modeling of myeloid sarcoma. Among the tested biomaterials, 5% GelMA emerged as the optimal candidate based on its mechanical properties, printability, oxygen permeability, and ability to support leukemic cell viability and spheroid formation *in vitro*. We used a GelMA‐based 3D culture system to investigate how the microenvironment regulates cell cycle dynamics, viability, and gene expression in myeloid leukemia cell lines. Finally, we assessed the transcriptional fidelity of GelMA‐induced spheroids by comparing them with primary myeloid sarcoma samples.

## Materials and methods

### Cell culture conditions

K562 cells were cultured in DMEM (Gibco‐Thermo Fisher Scientific, Waltham, MA, USA) supplemented with 10% fetal bovine serum, penicillin 100 U·mL^−1^, and streptomycin 100 μg·mL^−1^. KG‐1 cells were cultured in DMEM supplemented with 20% fetal bovine serum and antibiotic. MOLM‐13, MOLM‐14, THP‐1, PL‐21, and OCI‐AML3 cells were cultured in RPMI 1640 medium (Thermo Fisher Scientific) supplemented with 20% fetal bovine serum (FBS; Biowest, Nuaillé, France) and 1% penicillin–streptomycin. All cells were maintained at 37 °C in a humidified incubator with 5% CO_2_ and passaged every 2 days. The cell line panel was selected to cover complementary biological contexts within myeloid leukemia. MOLM‐13, MOLM‐14, OCI‐AML3, PL‐21, KG‐1, and THP‐1 were used as acute myeloid leukemia models, whereas K562 was included as a chronic myeloid leukemia model in blast crisis. PL‐21 was of particular interest because it was derived from an extramedullary myeloid sarcoma lesion, providing a relevant cellular background for modeling tissue‐associated leukemic growth. This panel allowed us to compare acute and chronic leukemia‐derived phenotypes and assess whether GelMA 5% could support spheroid formation across biologically diverse myeloid leukemia models. Detailed characteristics of the cell lines, including providers, research resource identifier (RRID), disease context, tissue of origin, lineage features, and main cytogenetic or molecular alterations, are provided in Table S1.

All cell lines used in this study were obtained from authenticated commercial cell banks (ATCC and DSMZ) and were authenticated by the respective providers within the past 3 years. Their identities were regularly verified against the corresponding reference profiles, by morphological criteria and immunophenotyping. All cell lines were confirmed to be free from cross‐contamination and mycoplasma contamination before experimental use.

### Hydrogel preparation and mechanical characterization

Six hydrogels were used: alginate cellulose (bioink), alginate cellulose with laminin α1β1γ1 (laminink 111), α4β1γ1 (laminink 411), α5β2γ1 (laminin 521), RGD peptides (Cellink RGD), and gelatin methacryloyl (GelMA). All the hydrogels were purchased from Cellink (Sweden). Alginate hydrogels were crosslinked with 50 mm CaCl_2_ for 10 min. GelMA was supplied at 20% (w/v) and prepared at 10% and 5% (w/v) in DPBS with 0.25% LAP as a photoinitiator. Polymerization was performed under visible light using a BioX6 LED at a distance of 4 cm for 30 s. The hydrogels were prepared at 37 °C before mixing with cells at a concentration of 3 × 10^6^ cells/mL. Stiffness measurements were performed using a JPK NanoWizard 3 Ultra or Bruker Resolve AFM with bright‐field imaging. Soft pyramidal probes were functionalized with a 1% Pluronic solution for 15 min and rinsed with Milli‐Q water to minimize hydrogel adhesion. The cantilever spring constants were calibrated using the thermal noise method. Elasticity measurements were performed in DMEM at 37 °C using the force mapping mode: 500 pN force setpoint, tip velocity of 5 μm·s^−1^, and 8 × 8 force curves per map over a 15–30 μm^2^ scan area. Measurements were taken from 10 areas and were repeated three times. Force‐distance curves were analyzed using jpk software with the Hertz model for a pyramidal indenter (35° half‐angle, Poisson's ratio 0.5). Data were visualized using r version 4.4.2 and ggplot2.

### Scanning electron microscopy (SEM) of hydrogel microstructure

The hydrogels were fixed with 1% glutaraldehyde in 0.1 m sodium cacodylate buffer overnight. After washing, the cells were treated with 1% osmium tetroxide in water for 1 h. The samples were dehydrated using increasing concentrations of ethanol, followed by baths in pure ethanol and hexamethyldisilazane. The hydrogels were dried overnight in a hood and mounted on stubs for observation using a Merlin Compact VP SEM (Zeiss, Oberkochen, Germany) at 1 kV voltage.

### 3D bioprinting and droplet‐based deposition

**Fig. 1:**
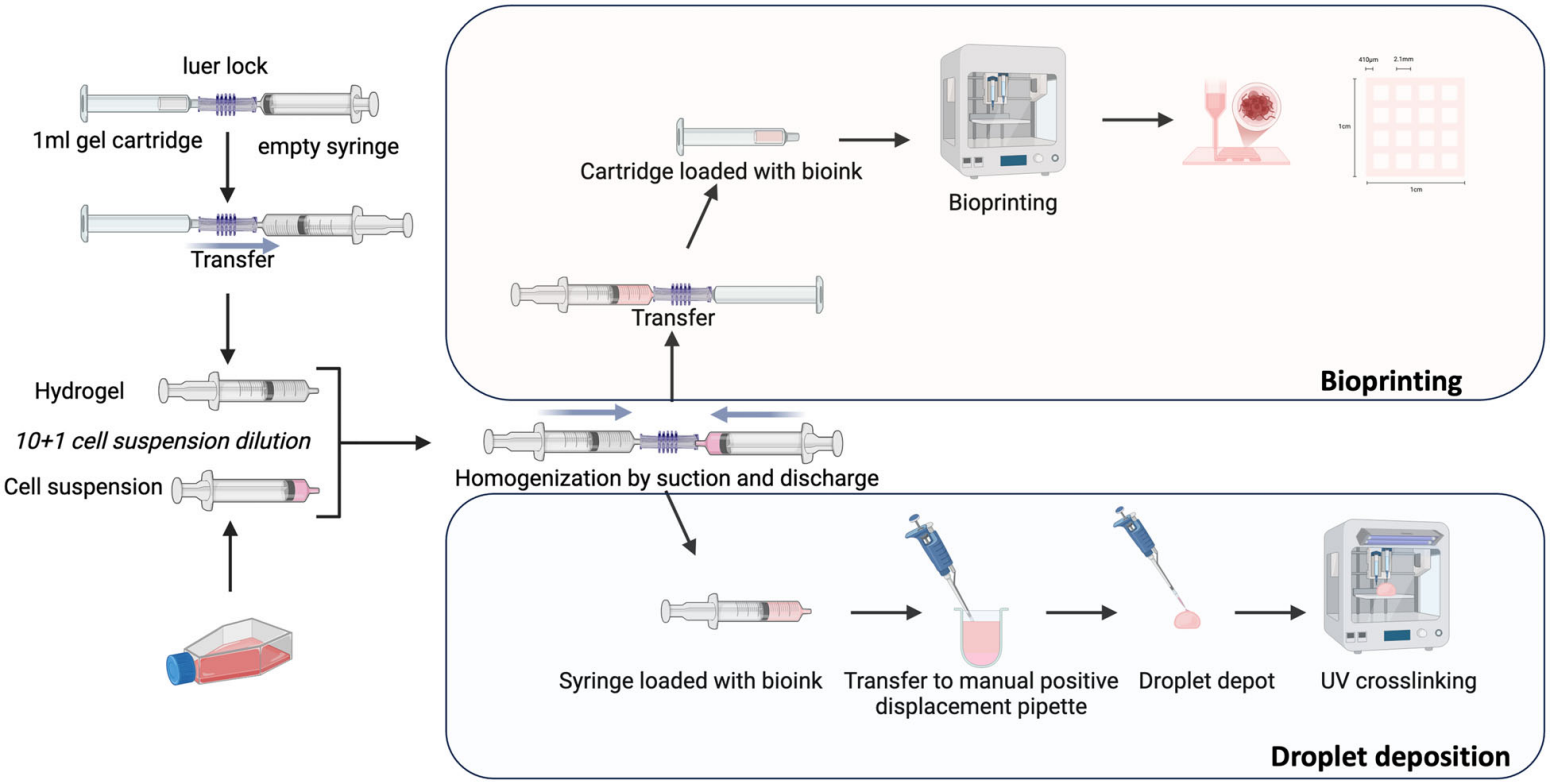
Workflow for bioink preparation and hydrogel structuring via bioprinting and droplet deposition. Cells cultured in monolayers were harvested and resuspended in culture medium. A 10 : 1 dilution of the hydrogel to cell suspension was performed using a double‐syringe system connected by a luer lock, enabling homogenization by repeated suction and discharge. The resulting bioink was either transferred into a printing cartridge for automated 3D bioprinting (top right) or a manual positive displacement pipette for droplet‐based deposition (bottom right). In both workflows, the final constructs were crosslinked under UV light to stabilize the hydrogel structure. Created in biorender. Germain, N. (2026) https://BioRender.com/q9t2k0s.

Bioprinting was performed using BioX and BioX6 extrusion‐based pneumatic 3D printers (Cellink). A standard pneumatic toolhead was used for nontemperature‐sensitive bioinks, whereas a temperature‐controlled toolhead was used for temperature‐sensitive bioinks. Cell suspensions in culture media were mixed with bioink at a 1 : 10 ratio, achieving a concentration of 3 × 10^6^ cells/mL (Fig. 1). The mixture was then transferred to UV‐shielded cartridges. For the GelMA bioink, the cartridges were preheated at 32 °C for 10 min until liquefaction was confirmed (Table 1). After cell addition, the printhead temperature was set to 18 °C, with a 12‐min equilibration at room temperature and 3‐min stabilization before printing. The constructs were printed as a 1 cm^2^ square grid pattern with 40% infill onto six‐well plates. For droplet deposition, the mixture was manually dispensed using a Gilson Microman E‐pipette. Crosslinking was performed under 405 nm light for 30 s at a distance of 4 cm from the resin surface. The constructs were covered with culture medium and incubated at 37 °C in 5% CO_2_ for 3 weeks.

**Table 1:** Printing and crosslinking parameters used in this study.

Biomaterial				Bioink parameters			Printing parameters			Crosslinking	
Base material	Adhesion protein	Commercial name	Viscosity at 25 °C	Biomaterial temperature	Biomaterial/cells mixing ratio	Number of cells in 1 ml bioink	Bioink temperature	Pressure (used [interval tested] in kPa)	Nozzle (gauge)	Method	Parameters
Alginate and nanofibrillated cellulose	None	Cellink Bioink	≥ 7 kPa·s at 0.01 s^−1^, ≤ 3 Pa·s at 200 s^−1^	RT	10 : 1	3 million	RT	7 [6,9]	22	Ionic	50 mm CaCl_2_ 90 s immersion
	Laminin α‐1, β‐1 and γ‐1	Cellink Laminink 111	≥ 5 kPa·s at 0.01 s^−1^, ≤ 3 Pa·s at 200 s^−1^	RT	10 : 1	3 million	RT	10 [8,11]	22	Ionic	
	Laminin α‐4, β‐2 and γ‐1	Cellink Laminink 411	≥ 5 kPa·s at 0.01 s^−1^, ≤ 3 Pa·s at 200 s^−1^	RT	10 : 1	3 million	RT	8 [7,10]	22	Ionic	
	Laminin α‐5, β‐2 and γ‐1	Cellink Laminink 521	≥ 5 kPa·s at 0.01 s^−1^, ≤ 3 Pa·s at 200 s^−1^	RT	10 : 1	3 million	RT	9 [8,10]	22	Ionic	
	Arginylglycylaspartic acid peptide motif	Cellink RGD	≥ 7 kPa·s at 0.01 s^−1^, ≤ 3 Pa·s at 200 s^−1^	RT	10 : 1	3 million	RT	15 [14,20]	22	Ionic	
Methacrylated Gelatin	None	Cellink GelMA	168 ± 50 Pa·s	32 °C	10 : 1	3 million	18 °C	12 [7,15] (GelMA 5%)	22	Physical (photocrosslinking)	UV 405 nm, 30 s at 3 cm of the center of the construct

### Pore structure and printability assessment

Pore printability was assessed using digital photographs captured on days 1–21. Five equal regions of interest were selected per image to measure the pore perimeter and area. The pore printability index (Pr) was calculated as Pr = (perimeter^2^)/(16 × area), where 1.0 indicates a perfect square and values approaching 0.0 indicate increasingly elongated polygons.

### Oxygen diffusion analysis in GelMA constructs

Oxygen tension within the GelMA hydrogels was measured using the OxyLite Pro system (Oxford Optronix, Adderbury, UK), a fiber‐optic platform based on fluorescence quenching technology that provides real‐time noninvasive measurements of oxygen partial pressure (pO_2_). The measurements were performed at room temperature, under static conditions. Hydrogels at 5%, 10%, and 20% GelMA concentrations were pre‐equilibrated in the culture medium, and fiber‐optic probes (500 μm tip diameter) were inserted into the center of each construct. Three measurements per hydrogel type were recorded, yielding 183 data points per condition with raw data in mmHg. To estimate the oxygen content as an atmospheric pressure percentage, the following formula was applied: %O_2_ = (pO_2_/760) × 100 [18].

### Supernatant biochemical and cell leakage analysis

Cell counts were performed using a Luna‐FL counter (Logos Biosystems, Villeneuve d' Ascq, France). Bioink supernatants were collected on days 1, 4, and 12. Cells were mixed 1 : 1 with trypan blue on Luna counting slides for the automated counting of total, live, and dead cells. Biochemical analyses were performed using the Cobas 8000 Analyzer (Roche, Basel, Switzerland). Glucose, lactate dehydrogenase (LDH), and lactate levels were measured using a spectrophotometer (Cobas 8000 Module C 701). The pH was measured using a Seven Compact S210 meter (Mettler‐Toledo, Greifensee, Switzerland).

### Histological and immunophenotypic analyses of PL21 and K562 GelMA spheroids

Immunohistological analyses were performed at the Pathology Department of Lille University Hospital (CHU de Lille) using standard, validated routine procedures. The spheroids were fixed, paraffin‐embedded, sectioned, and stained with HES. Immunohistochemistry was performed using antibodies against myeloperoxidase (MPO), CD34, CD43, CD45, and CD117, and the staining patterns were qualitatively compared with those of representative patient‐derived myeloid sarcoma samples (*n* = 3).

### Cell viability, morphometry, and imaging analyses

The hydrogels were stained with calcein‐AM, Propidium Iodide, and Hoechst 33342 (Thermo Fisher Scientific) to label live and dead cells and nuclei on days 1, 5, 12, and 21. After rinsing with PBS, the cells were incubated with the staining solution for 30 min at 37 °C in 5% CO_2_, washed, and covered with culture medium. Imaging was performed using an EVOS FLoid microscope on day 1 and a Zeiss Yokogawa CSU‐X1 microscope on days 5, 12, and 21, respectively. On day 1, viability was counted in 10 ROIs per sample across the three images and expressed as cells/mm^2^. On days 5, 12, and 21, six spheroids were analyzed per condition. Viability = (green intensity)/(green intensity + red intensity) normalized to the spheroid size. Spheroid number, size, and circularity were analyzed using fiji software, with circularity = 4pi(area/perimeter^2^) [19]. For cytoskeletal analysis on days 12 and 21, samples were fixed with 4% PFA, stained with Rhodamine Phalloidin and Hoechst 33342 33 342 (Thermo Fisher Scientific), and imaged using a Zeiss Yokogawa CSU‐X1 microscope. Images were processed using zen blue Software 3.12 (Zeiss, Oberkochen, Germany) and imagej software [20].

### Cell cycle analysis by flow cytometry

Cell cycle distribution was analyzed in suspension cultures and 5% GelMA hydrogels on days 1, 5, 12, and 21 using propidium iodide (PI) staining [21]. Flow cytometry was performed using CytoFLEX LX (Beckman‐Coulter, Brea, USA) and analyzed using kaluza analysis Software 2.1 (Beckman‐Coulter) to determine the cell cycle phase percentages.

### EdU incorporation assay after GelMA release

To assess whether cells recovered from GelMA 5% spheroids could re‐enter the cell cycle after release from the 3D matrix, 5‐ethynyl‐2′‐deoxyuridine (EdU) incorporation was analyzed by flow cytometry after spheroid dissociation and replating at the indicated times using the Invitrogen Click‐iT EdU cell proliferation assay (Invitrogen, Carlsbad, CA, USA). Briefly, GelMA 5% constructs were recovered from 12‐well plates, the culture medium was removed, and gels were digested with 1 mL per well of collagenase type I at 2 mg·mL^−1^ for 25 min at 37 °C. The released cells were collected, counted, and their viability was assessed using trypan blue exclusion on a LUNA automated cell counter. The cells were then centrifuged at 400× ***g*** for 5 min, resuspended in their respective standard culture media, and replated in 12‐well plates at a density of 1 × 10^6^ cells/mL. At the indicated time points after replating, corresponding to T0, T24, and T48, the cells were incubated with 10 μm EdU for 1 h 30 min at 37 °C. The cells were then collected, centrifuged at 400× ***g*** for 5 min, and washed with PBS containing 1% BSA. After fixation with 4% PFA for 15 min at room temperature, the cells were washed and permeabilized with 0.5% Triton X‐100 for 15 min at room temperature. EdU detection was performed using a Click‐iT reaction cocktail containing 1× Click‐iT reaction buffer, copper protectant, Alexa Fluor 488 azide, and a reaction buffer additive. Cells were incubated for 30 min at room temperature in the dark, washed with PBS, and analyzed by flow cytometry using a Fortessa X20 cytometer. EdU‐positive cells were quantified to evaluate DNA synthesis recovery and cell cycle re‐entry after release from GelMA 5% spheroids.

### Reversibility assay after GelMA release

To assess the reversibility of the GelMA‐induced growth‐restricted phenotype, GelMA 5% spheroids were enzymatically dissociated and replated under standard 2D suspension culture conditions. Briefly, spheroids were recovered from the GelMA constructs, dissociated using collagenase type I, washed, and viable cells were counted using trypan blue exclusion with an automated cell counter. The cells were then replated at the same initial viable cell density in a standard culture medium. Viable cell numbers were quantified at 0, 24, and 48 h after replating. The results were expressed as fold changes relative to T0 after replating and as absolute viable cell numbers.

### RNA extraction and quality control

RNA was extracted from day 15 samples using a Quick‐RNA Microprep Kit (Zymo Research, Irvine, CA, USA) after cell dissociation with collagenase type I. RNA quality was assessed using 260/280 and 260/230 ratios (NanoDrop spectrophotometer; Thermo Fisher Scientific) and integrity was evaluated using an RNA 6000 Nano Kit on an Agilent 2100 Bioanalyzer (Agilent Technologies, Santa Clara, CA, USA). Samples with an RNA Integrity Number > 7 were selected and stored at −80 °C before shipping to Singleron Biotechnologies (Koln, Germany) for sequencing.

### RNA sequencing and library preparation

RNA was processed using an AccuraCode RNA‐Seq Kit (cat. no. 10710174; Singleron Biotechnologies GmbH) according to the manufacturer's protocol. Polyadenylated mRNA was isolated using AccuraCode beads (Cat. no. 1200030080; Singleron). cDNA was generated with sample barcodes and UMIs, pooled, amplified for library preparation, and sequenced on an Illumina NovaSeq X using paired‐end 150 bp reads.

### RNA‐seq data processing and differential expression analysis

Raw sequencing reads were processed using CeleSCOPE v2.5.0‐dev and the multi_bulk_rna pipeline (https://github.com/singleron-RD/CeleScope). Differential gene expression analysis between myeloid sarcoma (MS) and suspension (SUS) cultures was performed using the DESeq2 package (v1.38.3) in R (v4.2.2) [22]. Eight samples were analyzed: four from 5% GelMA‐based spheroids (MOLM‐13, PL‐21, K562, and OCI‐AML3) and four from the suspension culture. The DESeq2 model used ‘SUS’ as the reference level. Genes with an adjusted *P*‐value (*P*adj) < 0.05 were considered significantly differentially expressed. Volcano plots highlighted genes with |log_2_FoldChange| > 1 and *P*adj < 0.05. Heatmaps of the top 30 upregulated and downregulated genes were created using the variance‐stabilizing transformation of the count data.

### Gene set and functional enrichment analyses

Gene set enrichment analysis (GSEA) was performed using the clusterprofiler package (v4.14.4) with 1000 permutations of genes ranked by log_2_ fold change [23]. Annotation was performed using org.Hs.eg.db, and hallmark gene sets from MSigDB v10.0 were retrieved using the msigdbr R package [24]. A separate enrichment analysis using the MatrisomeDB gene set was conducted to explore ECM‐related signatures [25]. Gene ontology (GO) enrichment was also performed on significantly upregulated and downregulated genes (*P*adj < 0.1), and the results were visualized by their functional categories. To assess the relevance of the 3D MS model, VST‐normalized expression data from local spheroids and suspension cultures were integrated with publicly available MS RNA‐seq data (GSE273877). Batch effects were corrected using the remote BatchEffect() function from the limma package. Principal component analysis (PCA) was conducted on the 500 most variable genes to evaluate the global transcriptional relationships. To highlight ECM‐specific programs, a focused heatmap was generated using the 30 most variable ECM‐related genes (GO:0031012), selected via biomaRt and filtered by the expression variance.

### Statistical analyses

The results in the bar graphs are expressed as the mean ± SD using graphpad prism 10.5 (www.graphpad.com; GraphPad Software, Boston, Massachusetts, USA). The groups were compared using one‐way ANOVA with Tukey's test or two‐way ANOVA with Geisser–Greenhouse correction. Statistical significance was set at *P* ≤ 0.05, with nonsignificant unlabeled bars. The reproducibility of spheroid formation was analyzed using intra‐ and interseries coefficients of variation (CV). Bartlett's test was used to assess variance homogeneity, and the Shapiro–Wilk test with Q–Q plots confirmed data normality.

## Results

### Hydrogel selection based on printability and structural properties

**Fig. 2:**
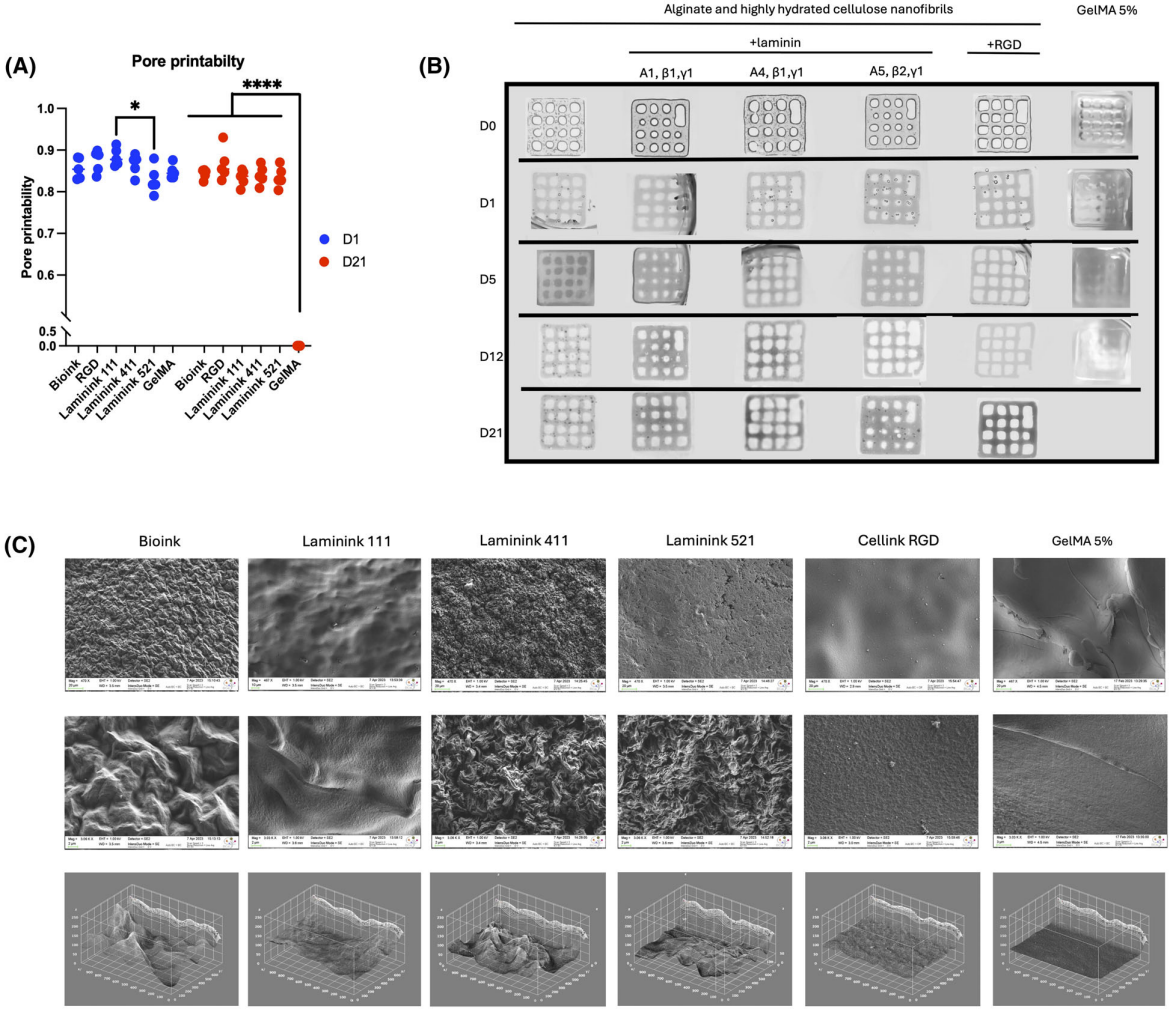
Structural properties and printability of candidate hydrogels (A) Representative scanning electron microscopy (SEM) micrographs of printed hydrogels showing differences in surface topography among alginate‐based matrices and methacrylated gelatin 5% (GelMA 5%) upper panel SEM micrographs acquired at low magnification (×470, scale bar: 20 μm) reveal the overall surface morphology of each hydrogel type, showing variability in roughness and fiber density. Middle panel: Higher magnification images (×3060, scale bar: 2 μm) highlight the microstructural differences, such as fiber orientation, porosity, and texture, between the hydrogels. Lower panel: Three‐dimensional surface plots generated from SEM images provide a comparative visualization of topographical irregularities and surface complexity. Together, these analyses illustrate the diversity of physical surface features among the tested biomaterials, which may influence cell–matrix interactions in 3D culture systems. (B) Temporal evolution of the pore printability index (Pr) in the hydrogels from days 1 to 21 (*n* = 5 independent samples per condition; two‐way ANOVA followed by Šídák's multiple‐comparisons test; *P < 0.05 and ****P < 0.0001). (C) Macroscopic degradation pattern of GelMA constructs over time compared with that of stable alginate‐based hydrogels.

To identify a suitable hydrogel for modeling myeloid sarcoma, we first evaluated six biomaterials for printability, structural fidelity, and mechanical properties. All hydrogels were printed using standardized extrusion settings. GelMA 5% achieved good shape fidelity and resolution, as indicated by the initial pore printability values (Pr ~ 0.85), which were comparable to those of the alginate‐based counterparts (Fig. 2A). However, GelMA underwent controlled degradation over time, losing its macroscopic structure after day 5, in contrast to alginate‐based hydrogels, which retained their geometry (Fig. 2B). SEM analysis revealed substantial surface heterogeneity across the alginate‐based matrices, with roughness often exceeding 200 μm. In contrast, GelMA 5% exhibited a smooth and uniform topography (Fig. 2C), consistent with low‐viscosity extrusion and homogeneous photocrosslinking.

### Mechanical and diffusion profiling of GelMA reveals favorable niche‐like properties

**Fig. 3:**
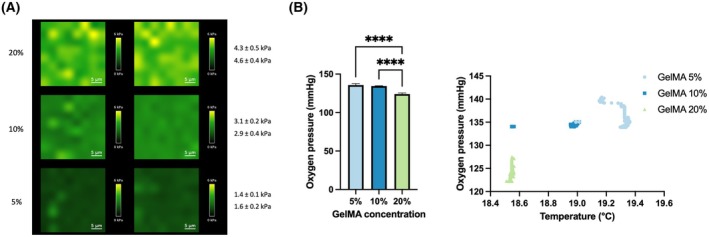
Biophysical profiling of GelMA hydrogels: Stiffness and oxygen permeability. (A) Atomic force microscopy (AFM)‐derived Young's modulus of GelMA at 5, 10, and 20% concentrations (*n* = 3, mean +/− SD, scale bar: 5 μm). (B) Partial oxygen pressure (pO_2_, mmHg) measured at the hydrogel center converted to %O_2_ relative to atmospheric pressure (*n* > 9, mean +/− SD, one‐way ANOVA followed by Tukey's multiple‐comparisons test; ****P < 0.0001).

Atomic force microscopy (AFM) characterization of hydrogels is crucial for developing biomimetic 3D culture systems that accurately recapitulate the physical microenvironment of myeloid sarcomas. AFM analysis demonstrated that the stiffness of GelMA scaled with the polymer concentration. GelMA 5% exhibited elastic moduli between 1.4 and 1.6 kPa, remarkably similar to that of soft tissues, while 10% and 20% concentrations yielded stiffer matrices exceeding 3 and 4.5 kPa, respectively (Fig. 3A). In parallel, oxygen diffusion was inversely correlated with stiffness: GelMA 5% maintained the highest partial oxygen pressure (~ 134.5 mmHg, ~ 17.7% atmospheric O_2_), whereas GelMA 20% dropped to 124.1 mmHg (~ 16.3%) (Fig. 3B). These data highlight GelMA 5% as the optimal balance between structural integrity, mechanical compliance, and oxygen permeability for sustained culture of leukemic cells.

### GelMA 5% maintains viability and promotes reproducible spheroid formation

**Fig. 4:**
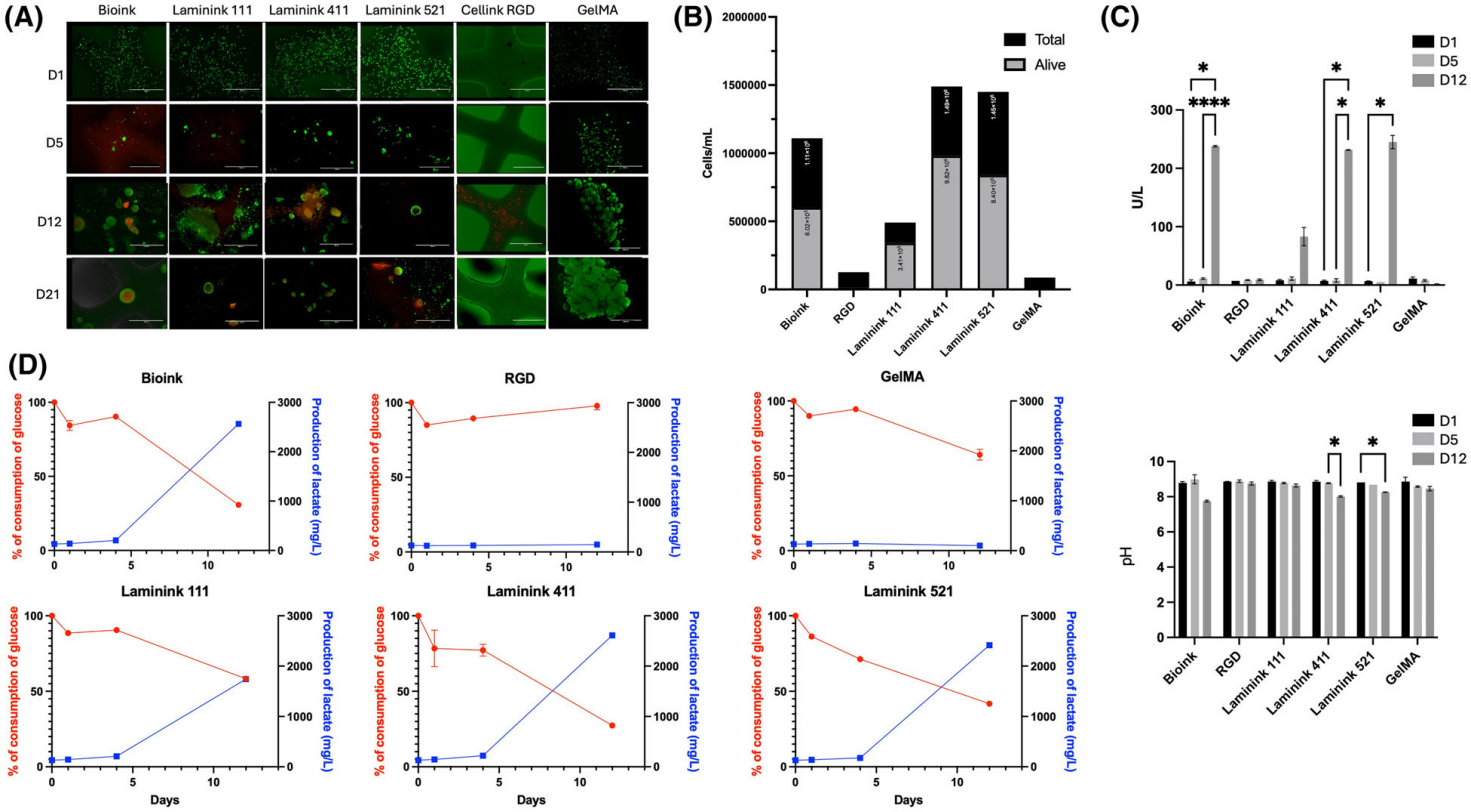
Cytocompatibility and metabolic profiling of encapsulated leukemic cells. (A) Fluorescence‐based live/dead cell viability imaging on days 1, 5, and 12 (scale bar: 1000 μm). (B) Cell leakage. Total and viable cell counts in the supernatants on day 12 (results representative of 1 of 3 independent experiments) (C) Glucose consumption, lactate production, and pH of the supernatants over 12 days (*n* = 2, mean +/− SD, two‐way repeated‐measures ANOVA followed by Šídák's multiple‐comparisons test; *P < 0.05 and ****P < 0.0001). (D) Quantification of lactate dehydrogenase (LDH) released into the supernatant as a cytotoxicity marker (*n* = 2, mean +/− SD, two‐way repeated‐measures ANOVA followed by Šídák's multiple‐comparisons test; *P < 0.05).

**Fig. 5:**
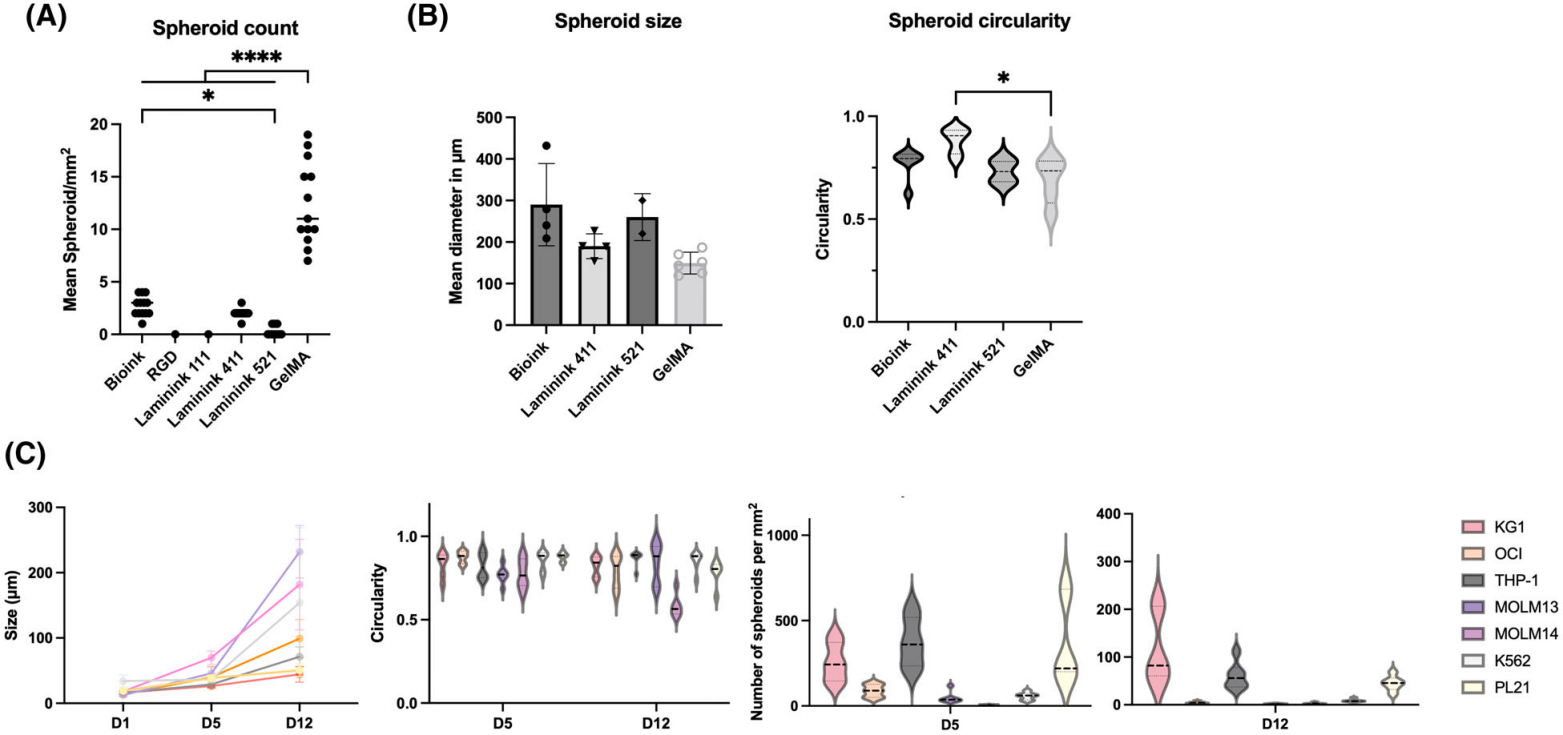
Spheroid formation capacity and morphometric analysis of GelMA. (A) Number of spheroids per mm^2^ on day 12 in different hydrogels using K562 cells (*n* > 6, one‐way ANOVA followed by Tukey's multiple‐comparisons test; *P < 0.05 and ****P < 0.0001). (B) Quantification of spheroid size (*n* ≥ 2, mean +/− SD, one‐way ANOVA followed by Tukey's multiple‐comparisons test; *P < 0.05) and circularity across GelMA‐ and alginate‐based matrices using K562 cells (*n* > 9, violin plots show the distribution of individual values; horizontal lines indicate the median and interquartile range). (C) Cell line‐specific spheroid size (*n* > 9, mean +/− SD), shape, and density metrics in GelMA 5% (day 12) (*n* > 9, violin plots show the distribution of individual values; horizontal lines indicate the median and interquartile range).

Next, we monitored cell viability to ensure the reliability of the hydrogel‐based cell culture. Early cell viability in GelMA was high (> 96%) and comparable across all hydrogels, except for RGD‐supplemented alginate (Fig. 4A). However, GelMA exhibited the lowest LDH release (2 U·L^−1^ on Day 12) and minimal cell leakage into the supernatant (~ 10^5^ cells/mL), indicating superior cytocompatibility and encapsulation fidelity (Fig. 4B,C). Glucose concentrations decreased over time in most hydrogels, reflecting active cellular metabolism (Fig. 4D). Similarly, lactate concentrations in the supernatant increased significantly over time in most hydrogels, indicating anaerobic metabolism and the potential onset of hypoxia within the constructs. Spheroid formation by K562 cells was observed from day 12 in multiple hydrogels, with GelMA supporting significantly more spheroids (12.46 ± 3.97 spheroids/mm^2^) than Bioink, Laminink 411, or Laminink 521 (*P* < 0.0001) (Fig. 5A). While GelMA yielded the highest spheroid count, Laminink 411 produced more circular spheroids (circularity 0.88 ± 0.06 vs. 0.69 ± 0.11 for GelMA, *P* < 0.05) (Fig. 5B). All seven myeloid leukemia cell lines tested formed spheroids in GelMA 5%, albeit with cell line‐specific dynamics (Fig. 5C). MOLM‐13 and MOLM‐14 formed large irregular aggregates (~ 300 μm), whereas OCI‐AML3, K562, and PL‐21 formed small circular spheroids (circularity > 0.82). THP‐1 and KG‐1 cells exhibited a high initial spheroid density but reduced size, suggesting rapid aggregation and fusion. These differences reflect inherent proliferative and adhesive behaviors but were consistently supported by GelMA across replicates (interseries CV = 5.9%). These morphometric differences were partly aligned with the biological diversity of the panel, which included AML‐derived models, a CML blast crisis model, and the MS‐relevant PL‐21 cell line. Thus, GelMA 5% supported spheroid formation across distinct myeloid leukemia contexts while preserving cell line‐specific aggregation patterns.

### Histological and immunophenotypic validation of GelMA spheroids

**Fig. 6:**
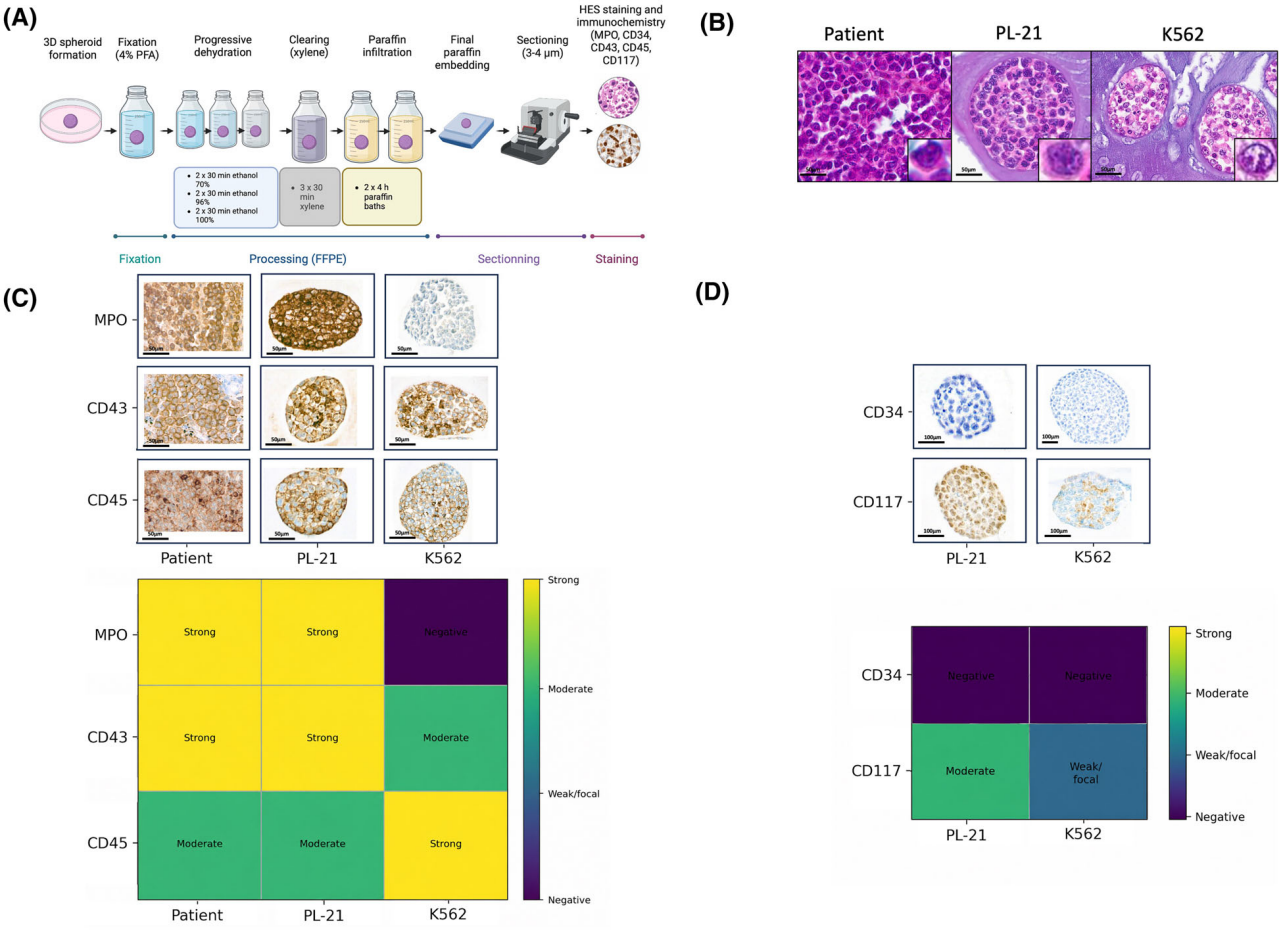
Histological and immunophenotypic comparison of PL21 and K562 GelMA spheroids with representative patient‐derived myeloid sarcoma samples. (A) Experimental workflow for histological and immunophenotypic comparison between GelMA‐based spheroids and clinical myeloid sarcoma samples. Created in biorender. Germain, N. (2026) https://BioRender.com/q9t2k0s (B) Hematoxylin–eosin–safran (HES) staining showed that PL21 and K562 GelMA spheroids formed dense cellular structures composed of large atypical blastoid cells with a high nucleus‐to‐cytoplasm ratio and prominent nucleoli, resembling the morphological features observed in patient‐derived myeloid sarcoma (original magnification ×400, scale bar: 50 μm). (C, D) Immunostaining for myeloperoxidase (MPO), CD34, CD43, CD45, and CD117, and semi‐quantitative heatmap summarizing the intensity. Direct comparison between patient tissue and spheroids was performed only for markers analyzed in both settings, namely MPO, CD43, and CD45 (C); (D) CD34 and CD117 staining was performed only on spheroids and was used for additional model characterization (scale bar: 100 μm).

To further assess the translational relevance of the GelMA‐based 3D model, PL‐21 and K562 GelMA spheroids were compared with a representative patient‐derived myeloid sarcoma (MS) sample obtained from a 56‐year‐old patient with colonic MS (Fig. 6A–C). Histological examination using HES staining revealed that both spheroid models reproduced the key blastoid features observed in the patient samples, including large atypical cells with a high nucleus‐to‐cytoplasm ratio, irregular nuclear contours, and prominent nucleoli (Fig. 6B). However, differences in tissue architecture were observed between the models. Patient‐derived MS tissue and K562 spheroids displayed denser and more compact cellular organization, whereas PL‐21 spheroids exhibited a comparatively looser architecture with lower overall cellular density. Immunohistochemical analyses further demonstrated the preservation of hematopoietic and myeloid‐associated phenotypic features within GelMA spheroids (Fig. 6C,D). The patient‐derived MS sample showed strong expression of MPO, CD43, and CD45, consistent with a myeloid phenotype. As commonly reported in the literature, CD34 and CD117 expression in MS is heterogeneous and may vary according to the differentiation status of tumor cells [26, 27]. PL‐21 spheroids closely reproduced this immunophenotypic profile, with strong MPO and CD43 expression, moderate CD45 positivity, absence of CD34 expression, and persistent CD117 staining. In contrast, K562 spheroids showed absent MPO staining, moderate CD43 and CD45 expression, and only weak focal CD117 positivity, consistent with the known erythroleukemic origin and less‐mature myeloid differentiation status of K562 cells. Overall, these findings indicate that GelMA‐based spheroids preserve cell line‐specific phenotypic characteristics while partially recapitulating the histological and immunophenotypic features of patient‐derived myeloid sarcomas. Among the tested models, PL‐21 spheroids displayed the closest phenotypic resemblance to patient‐derived MS samples, particularly in terms of myeloid marker expression. These results show that GelMA spheroids recapitulate the selected features of myeloid sarcoma while retaining cell‐line‐specific phenotypes.

### 3D encapsulation in GelMA induces G1 arrest and apoptotic progression

**Fig. 7:**
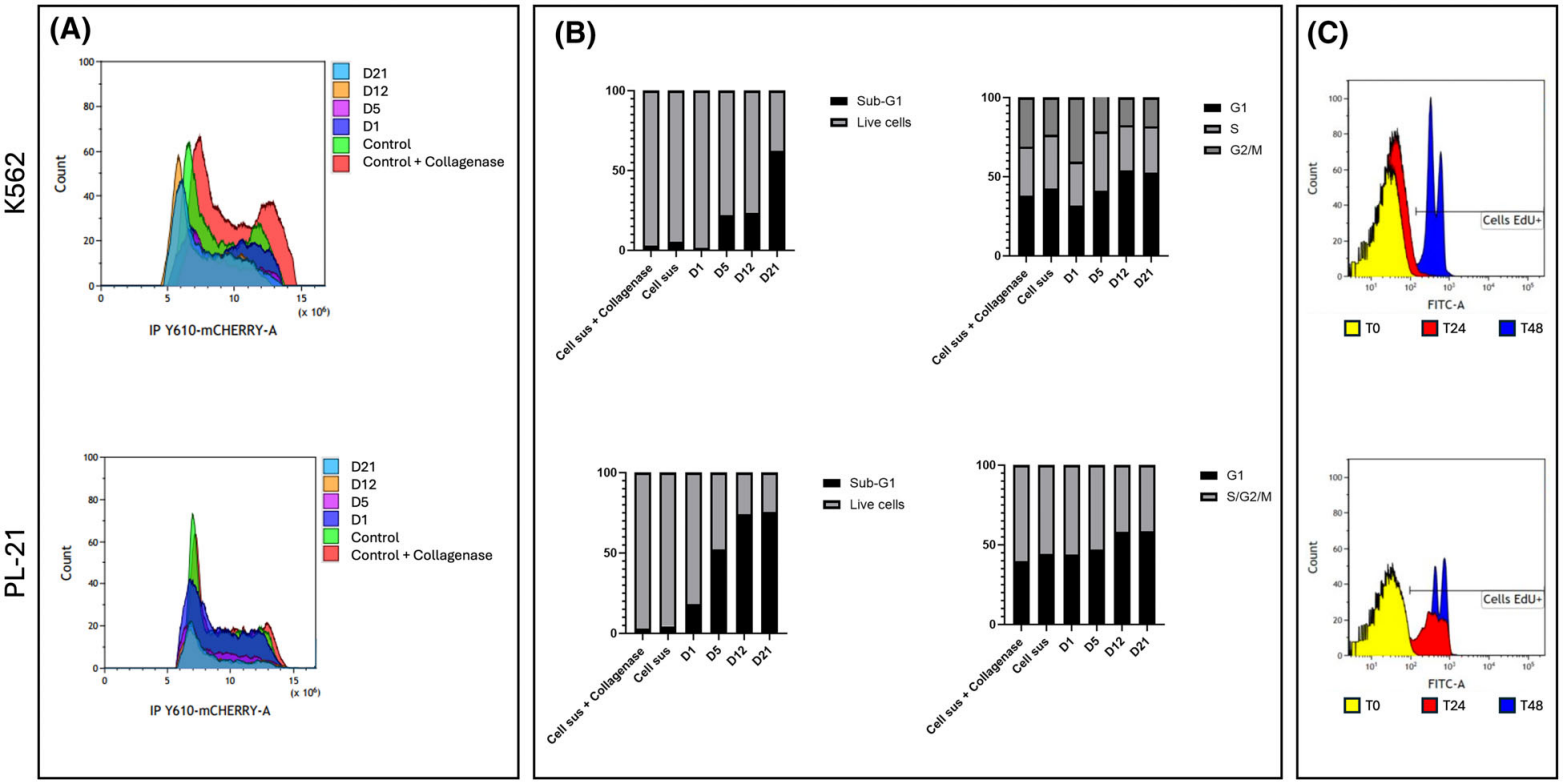
G1 arrest and apoptosis in 3D GelMA cultures. (A) Flow cytometry profiles showing progressive G1 enrichment and sub‐G1 accumulation in K562 and PL‐21 cells cultured in GelMA 5% for up to 21 d. (B) Percentage of cells in the G1, S, G2/M, and sub‐G1 phases over time in 3D versus suspension culture. (C) 5‐ethynyl‐2′‐deoxyuridine (EdU) incorporation after release from 5% GelMA spheroids and replating under standard 2D culture conditions. Representative flow cytometry histograms show EdU incorporation at T0, T24, and T48 after replating in K562 and PL‐21 cells, indicating progressive re‐entry into the S phase after release from the GelMA matrix.

To assess the functional consequences of spheroid formation, we analyzed the cell cycle progression of K562 and PL‐21 cells cultured in GelMA versus those cultured in suspension (Fig. 7A). Progressive accumulation in the G1 phase was observed in both lines, accompanied by a marked increase in sub‐G1 events, reaching 75% in PL‐21 by Day 21 (Fig. 7B). These results confirmed that the 3D GelMA microenvironment induces cell cycle slowdown and apoptosis, recapitulating the spatial and metabolic constraints of tissue‐infiltrating myeloid tumors.

To determine whether GelMA‐induced cell cycle arrest reflected an irreversible loss of proliferative capacity or a reversible quiescence‐like state, cells were released from 5% GelMA spheroids and replated under standard 2D culture conditions. EdU incorporation analysis showed that both K562 and PL‐21 cells were able to re‐enter the S phase after release, with detectable EdU‐positive cells at 24 h and a stronger EdU‐positive population at 48 h after replating (Fig. 7C). These findings indicate that GelMA encapsulation induces a reversible cell cycle restriction rather than a definitive proliferative arrest. Both K562 and PL‐21 cells progressively increased in number after release from GelMA, as shown by fold change analysis normalized to T0 and absolute viable cell counts (Fig. S1). Although GelMA‐derived cells expanded more slowly than cells maintained under standard 2D conditions, the recovery of viable cell expansion after removal from the 3D matrix supports that GelMA‐induced growth restraint is at least partially reversible (Fig. S1). Together, these results suggest that GelMA 5% encapsulation transiently restricts cell cycle progression through G1 arrest, potentially via spatial confinement and altered nutrient and oxygen diffusion, while preserving the proliferative potential of leukemic cells.

### RNA‐Seq confirms transcriptomic reprogramming in GelMA spheroids

**Fig. 8:**
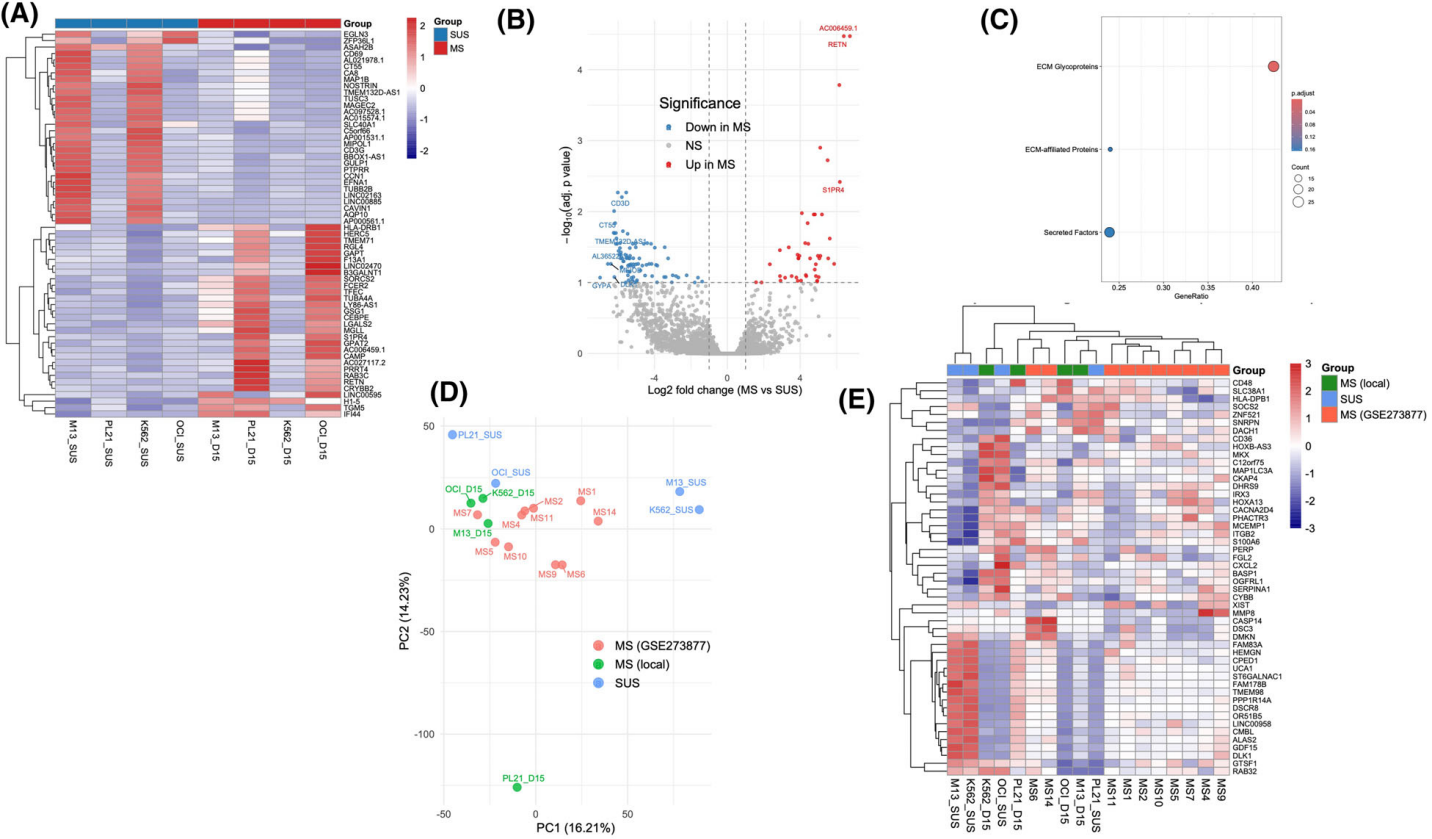
Transcriptomic reprogramming of leukemic cells in GelMA‐based 3D spheroids. (A) Heatmap of differentially expressed genes in leukemia cell lines (OCI‐AML3, K562, PL‐21, and MOLM‐13) cultured in suspension (SUS) or spheroids (MS). (B) Volcano plot showing significantly differentially expressed genes between MS and SUS conditions (adjusted *P*‐value < 0.05, log_2_ fold change threshold). Genes associated with inflammation, invasion, and lipid metabolism (MMP9, S1PR4, RETN, CXCL8, and FABP4) were highlighted. (C) Gene Set Enrichment Analysis (GSEA) of the matrisome gene set showing significant alterations in matrisome‐related pathways under MS conditions. (D) Principal component analysis integrating single‐cell profiles from MS spheroids and patient‐derived myeloid sarcoma samples (GSE273877). PL‐21 and MOLM‐13 spheroids clustered with patient cells. (E) Heatmap comparing the gene expression profiles of MS spheroids and myeloid sarcoma patient samples. The shared upregulation of extracellular matrix (ECM) and inflammatory genes confirmed transcriptional convergence.

To explore the impact of 3D culture on leukemic transcriptional profiles, we performed single‐cell RNA sequencing on four myeloid leukemia cell lines (OCI‐AML3, K562, PL‐21, and MOLM‐13) grown in standard 2D suspension (SUS) or as 3D spheroids that mimic myeloid sarcoma (MS). The heatmap (Fig. 8A) revealed robust transcriptional reprogramming under MS conditions, with multiple genes strongly upregulated in spheroids compared to those in suspension. Differential expression analysis (Fig. 8B, volcano plot) identified key genes upregulated in MS spheroids, including MMP9 (ECM remodeling), S1PR4 (sphingolipid signaling), RETN, CXCL8, CCL4 (inflammatory cytokines), FABP4, and CD36 (lipid metabolism), highlighting major changes in extracellular matrix interaction, immune signaling, and metabolic adaptation induced by 3D culture. To assess whether ECM‐related programs were enriched, we performed gene set enrichment analysis (GSEA) using the matrisome database (Fig. 8C). The analysis revealed a significant downregulation of extracellular matrix glycoproteins under MS conditions (NES = −1.76, *P* = 0.0002), whereas other categories, such as ECM‐affiliated proteins and secreted factors, were not significantly enriched. These results suggest the selective repression of specific ECM components rather than the global activation of adhesion or remodeling pathways. To evaluate the clinical relevance of these profiles, we integrated single‐cell data from patients with myeloid sarcoma (GSE273877) [28]. Principal component analysis (Fig. 8D) demonstrated that PL‐21 and MOLM‐13 spheroids clustered closely with patient‐derived MS cells, whereas 2D SUS‐cultured cells remained transcriptionally distinct from them. This was confirmed by the integrated heatmap (Fig. 6E), where MS spheroids and patient samples shared a high expression of a core set of inflammation‐ and ECM‐related genes. Together, these data suggest that 3D culture induces disease‐relevant programs that are absent in standard suspension models, thus validating the use of MS spheroids as tractable preclinical models for extramedullary myeloid sarcoma.

## Discussion

Myeloid sarcoma (MS) is a rare and complex extramedullary manifestation of acute myeloid leukemia (AML), often associated with unique stromal interactions, hypoxic microenvironments, and a gene expression profile distinct from intramedullary disease [1, 4]. Traditional two‐dimensional (2D) culture systems fail to mimic the structural and metabolic constraints of tissues. In this study, we demonstrated that 5% GelMA hydrogels provide a reproducible, biocompatible, and biologically relevant 3D platform for modeling the key features of MS using various leukemia cell lines.

We identified GelMA 5% as the optimal matrix among several candidate hydrogels based on its printability, smooth surface morphology, stiffness (~ 1.5 kPa), and oxygen permeability, which are critical parameters for replicating the bone marrow niche and extramedullary environments. Although alginate‐based hydrogels maintain longer shape fidelity, their coarse structure and limited cytocompatibility hinder spheroid formation and viability. In contrast, GelMA supported the consistent self‐assembly of myeloid leukemia cells into spheroids with minimal cell leakage and metabolic stress, underscoring its superior structural and biological performance.

Biologically, GelMA encapsulation induces G1‐phase accumulation and apoptosis in leukemic cells, supporting a growth‐restricted and dormant‐like phenotype reminiscent of extramedullary leukemic niches [29]. These findings are consistent with previous *in vivo* observations showing reduced cycling and increased susceptibility to apoptosis in MS lesions [7, 8, 30]. Importantly, the reversibility and EdU assays further indicated that this phenotype was not irreversible, as K562 and PL‐21 cells released from GelMA spheroids resumed viable cell expansion and demonstrated single‐cell S phase re‐entry after replating in standard 2D suspension culture. These data provide functional evidence that GelMA‐induced growth restraint is reversible and driven by microenvironmental constraints rather than a permanent loss of proliferative capacity.

Our findings demonstrate that the self‐organization of leukemic cells into spheroids within a 3D matrix is sufficient to induce extensive transcriptional reprogramming, even in the absence of stromal cells or exogenous signals [31, 32]. This effect may be primarily driven by biophysical and metabolic constraints imposed by the 3D microenvironment. The physiological stiffness of 5% GelMA (~ 1.5 kPa) closely mimics the mechanical properties of soft tissues commonly infiltrated by myeloid sarcomas, thereby modulating mechanotransduction and cytoskeletal organization. In parallel, limited oxygen diffusion within the hydrogel generates mild hypoxia, a well‐known driver of G1 cell cycle arrest, metabolic rewiring, and stress adaptation. Together, these conditions triggered the upregulation of genes involved in inflammatory signaling (CXCL8, CCL4, and RETN), matrix remodeling (MMP9), and lipid metabolism (FABP4, CD36). These results support the notion that the architecture and physical properties of the tumor microenvironment are potent regulators of the leukemic phenotype, independent of soluble cues or stromal coculture conditions. Thus, our 3D model recapitulates the critical features of extramedullary myeloid sarcoma through spatial organization alone, underscoring its value as a preclinical platform.

In the present study, we deliberately focused on soft‐tissue/extramedullary MS and used 5% GelMA, whose low stiffness range is more consistent with compliant soft tissues than with mineralized or periosteal niches. Therefore, our model should not be considered a direct mimic of bone‐associated MS, where leukemic blasts may be exposed to higher matrix stiffness, spatial confinement, compressive forces, and specific osteogenic cues. Future adaptations of this platform could address this limitation by increasing the GelMA concentration or developing hybrid bioinks incorporating PEGDA and/or nano‐hydroxyapatite to generate stiffer, bone‐like matrices. Such approaches would allow the investigation of how mechanical pressure, matrix rigidity, and mineralized cues regulate MS cell survival, proliferation, mechanotransduction pathways, and potential osteotropic behavior. In support of this strategy, Baruffaldi et al. showed that GelMA‐based composite hydrogels reinforced with nano‐hydroxyapatite and nanosilicate could be engineered as bone‐mimetic matrices for bone tissue applications, highlighting the tunability of GelMA in mineralized microenvironments [16].

Our panel included several AML‐derived cell lines, namely MOLM‐13, MOLM‐14, OCI‐AML3, PL‐21, KG‐1, and THP‐1, together with K562, a CML‐derived blast crisis model. This distinction is relevant because myeloid sarcoma is most commonly associated with AML, whereas CML‐derived models may display different adhesive, proliferative, and differentiation properties. Consistent with this heterogeneity, all leukemia cell lines formed spheroids in GelMA 5%, but with distinct morphometric behaviors. MOLM‐13 and MOLM‐14 formed large and irregular aggregates, whereas OCI‐AML3, K562, and PL‐21 generated smaller and more circular spheroids. THP‐1 and KG‐1 cells exhibited a high initial spheroid density with smaller structures, suggesting rapid aggregation and/or fusion dynamics. These differences likely reflect intrinsic lineage, maturation, genetic, and adhesive programs rather than a limitation of the GelMA platform. Importantly, PL‐21, derived from an extramedullary myeloid sarcoma lesion, represents a particularly relevant model for studying tissue‐associated leukemic growth. In transcriptomic analysis, PL‐21 and MOLM‐13 spheroids clustered closer to patient‐derived MS samples than their suspension counterparts, suggesting that the 3D GelMA environment can reveal disease‐relevant programs in selected AML backgrounds. In contrast, the inclusion of K562 cells broadened the model by demonstrating that GelMA also supports spheroid formation in a CML blast crisis context while preserving lineage‐specific features such as weak MPO and CD117 expression compatible with its erythroleukemic origin. Thus, the diversity of the panel should be viewed as a strength of the model, highlighting both its versatility and the need to interpret GelMA‐induced phenotypes in light of the acute versus chronic leukemia context.

We showed that 3D spheroid culture of myeloid leukemia cell lines induced transcriptional reprogramming of genes associated with inflammation, matrix remodeling, and metabolic adaptation. This transition, observed in PL‐21 and MOLM‐13, is driven by the upregulation of key effectors, such as MMP9, S1PR4, RETN, and FABP4, suggesting that 3D architecture mimics extramedullary signals capable of reactivating tissue‐invasive and immunomodulatory programs. S1PR4, which encodes a sphingosine‐1‐phosphate receptor, has been implicated in leukocyte trafficking via lymphatic routes and may enhance leukemic cell migration in 3D contexts [33]. RETN, which encodes the pro‐inflammatory cytokine resistin, is restricted to the myeloid lineage, is regulated by CEBPE, and has been proposed as a biomarker of chronic myeloid leukemia (CML) and adverse AML subtypes [34, 35, 36]. Gene set enrichment analysis using MatrisomeDB revealed significant downregulation of extracellular matrix glycoproteins in MS‐like spheroid cultures (NES = −1.76, *P* = 0.0002), whereas other matrisome‐related categories showed no significant enrichment, suggesting selective alteration rather than global activation of matrisome pathways. Lineage‐specific markers were suppressed and CD3D, which encodes a T‐cell receptor complex component linked to immune activation and treatment response in AML [37, 38], was downregulated. Similarly, GYPA, a marker of terminal erythroid differentiation repressed by HDAC6 and regulated by miR‐218 during leukemic reprogramming [39, 40, 41], was also downregulated. GYPA downregulation indicates a loss of erythroid differentiation potential, consistent with a shift toward an immature tissue‐invasive myeloid phenotype [42, 43]. These changes align with extramedullary leukemic dissemination, in which tumor cells navigate stromal‐rich tissue. The transcriptional profiles of the 3D‐cultured cells converged with those of the primary myeloid sarcoma samples, as shown by PCA and heatmap integration. This alignment validated the biological fidelity of the model and revealed shared signatures that reflected extramedullary tropism features. The PL‐21 cell line derived from myeloid sarcoma maintained a transcriptomic identity aligned with patient‐derived profiles under 3D conditions, highlighting its potential as a preclinical tool.

Transcriptomic comparisons with patient‐derived MS samples (GSE273877) showed that spheroids derived from PL‐21 and MOLM‐13 cells closely recapitulated *in vivo* profiles, reinforcing the clinical relevance of our 3D system. PL‐21, originally isolated from an MS lesion, has emerged as a reliable model, retaining high expression of MS‐associated transcripts, such as CXCL8, MMP9, and FABP4, under GelMA conditions.

Collectively, these results establish that 5% GelMA is a robust and flexible matrix that induces spheroid formation, preserves cellular viability, and triggers a physiologically meaningful transcriptomic state that mirrors patient‐derived MS. By integrating physical, phenotypic, and molecular evidence, this study fills a critical gap between 2D culture and *in vivo* models of extramedullary acute leukemia.

## Conclusion

This study validated GelMA 5% as a physiologically relevant platform for modeling the architecture and biology of myeloid sarcomas *in vitro*. Through a combination of structural fidelity, mechanical compliance, and biocompatibility, GelMA hydrogels enable leukemic cells to self‐organize into viable spheroids that reflect the key features of extramedullary leukemia, including reversible quiescence, apoptosis, and transcriptomic reprogramming. Our findings suggest that this 3D system is a promising preclinical tool for studying extramedullary leukemogenesis, identifying therapeutic vulnerabilities, and screening agents targeting dormancy, invasion, and metabolic stress responses in MS.

Future developments integrating stromal cells, immune components, and vascular perfusion could further enhance physiological fidelity and translational impact. Ultimately, this model paves the way for personalized medical platforms that can mimic extramedullary dissemination.

## Conflict of interest

The authors declare that they have no affiliations with or involvement in any organization or entity with any financial interest in the subject matter or materials discussed in this manuscript.

## Author contributions

NG and P. Marchetti conceived and supervised the study; NG, LHTN, SL, EB, and P. Marchetti designed the experiments; NG, LHTN, NT, MD, MC, SJ, EB, and PM performed the experiments; NG, LHTN, EB, PM, SL, RD, and P. Marchetti analyzed the data; NG and P. Marchetti wrote the manuscript.

## Supporting information


**Fig. S1.** Recovery of viable cell expansion after release from the GelMA spheroids. (A) Experimental workflow. K562 and PL‐21 GelMA 5% spheroids were enzymatically dissociated and replated under standard 2D suspension culture conditions. Viable cell counts were performed at 0, 24 and 48 h; Created in biorender. Germain, N. (2026) https://BioRender.com/q9t2k0s (B) Viable cell expansion expressed as fold change relative to T0 after replating (*n* = 3, mean +/− SD); (C) Absolute viable cell numbers after replating. GelMA‐derived cells progressively increased in number after release from the 3D microenvironment, supporting the partial reversibility of the GelMA‐induced growth‐restricted phenotype (*n* = 3, mean +/− SD two‐way ANOVA, with experimental condition and time as factors, followed by Šídák's multiple‐comparisons test). This assay provides population‐level evidence of recovery but does not directly assess S‐phase entry at the single‐cell level.
**Table S1.** Characteristics of the cell lines used in this study (Species, cell type, tissue of origin, and cytogenetic features).

## Data Availability

RNA‐sequencing data generated in this study were deposited in the ArrayExpress Data Depository (https://www.ebi.ac.uk/biostudies/arrayexpress) with the series accession number E‐MTAB‐15359. Raw data are available from the corresponding author upon reasonable request.
