# Supplementary material for: GelMA‐based 3D spheroids recapitulate transcriptomic and functional hallmarks of myeloid sarcoma
Source: FEBS Open Bio. 2026 Aug 13:10.1002/2211-5463.70321. Online ahead of print. doi: 10.1002/2211-5463.70321 (PMC13470959; doi:10.1002/2211-5463.70321)
Supplement: Supplementary file 1 [file FEB4-9999-0-s001.docx]

**Supporting information**

| Cell line | MOLM14 | MOLM13 | THP-1 | PL21 | K562 | KG1 | OCI-AML3 | Jurkat, Clone E6-1 |
| --- | --- | --- | --- | --- | --- | --- | --- | --- |
| Provider /  Identification number | DSMZ ACC 777 | DSMZ ACC 554 | ATCC TIB-202 | DSMZ ACC 536 | ATCC CCL-245 | ATCC CCL-246 | DSMZ ACC 582 | ATCC TIB-152 |
| RRID | CVCL_7916 | CVCL_2119 | CVCL_0006 | CVCL_2161 | CVCL_0004 | CVCL_0374 | CVCL_1844 | CVCL_0367 |
| Specie | Human | Human | Human | Human | Human | Human | Human | Human |
| Cell type | Acute Myeloid Leukemia | Acute Myeloid Leukemia | Acute Myeloid Leukemia | Acute Myeloid Leukemia | Chronic Myeloid Leukemia | Acute Myeloid Leukemia | Acute Myeloid Leukemia | Acute Lymphocityc Leukemia |
| Origin | established from the peripheral blood of a 20-year-old man with acute myeloid leukemia AML FAB M5a at relapse in 1995 after initial myelodysplastic syndrome (MDS, refractory anemia with excess of blasts, RAEB); carries internal tandem duplication of FLT3; cell line carries the CBL deltaExon8 mutant; sister cell line of MOLM-13 (ACC 554) | established from the peripheral blood of a 20-year-old man with acute myeloid leukemia AML FAB M5a at relapse in 1995 after initial myelodysplastic syndrome (MDS, refractory anemia with excess of blasts, RAEB); described to carry internal tandem duplication of FLT3; the FLT3 protein is not expressed; cell line carries the CBL deltaExon8 mutant; sister cell line of MOLM-14 (ACC 777). | established from the peripheral blood of a 1-year-old boy with acute monocytic leukemia (AML) at relapse in 1978; the cells can be used for induction of differentiation studies; the cells were described in the literature to produce lysozyme and to be phagocytic; carries t(9;11)(p21;q23) leading to KMT2A-MLLT3 (MLL-MLLT3; MLL-AF9) fusion gene. | established from the peripheral blood of a 24-year-old man with refractory acute promyelocytic leukemia after mediastinal granulocytic sarcoma; however, PL-21 cells do not carry the t(15;17); based on morphology, surface marker expression of CD14/CD68 and expression of the monocyte-specific esterase, PL-21 cells appear to be rather of monocytic origin; differentiation is inducible by phorpbol ester; cells carry an FLT3 internal tandem duplication | established from the pleural effusion of a 53-year-old woman with chronic myeloid leukemia (CML) in blast crisis in 1970; cells carry the BCR-ABL1 e14-a2 (b3-a2) fusion gene; this subclone (ACC 10) weakly expresses MHC class I (image); other subclones (ACC 86 and ACC 92) are MHC class I negative and might be used in in-vitro natural killer assays; cells produce hemoglobin. | established from the bone marrow of a 59-year-old man with erythroleukemia that developed into acute myeloid leukemia (AML) at relapse in 1977; cells were described in the literature to form colonies in soft-agar in response to colony-stimulating factor and to form dendritic-like cells (DLC) in response to GM-CSF, IL-4, PMA (TPA), and ionomycin; sister cell line KG-1a | established from the peripheral blood of a 57-year-old man with acute myeloid leukemia (AML FAB M4) at diagnosis in 1987; cells carry an NPM1 gene mutation (type A) and the DNMT3A R882C mutation. | Jurkat, Clone E6-1 is a clone of the Jurkat-FHCRC cell line, a derivative of the Jurkat cell line, which was established from the peripheral blood of a 14-year-old, male, acute T-cell leukemia patient. |
| Cytogenetics | human hyperdiploid karyotype; -49(46-50)<2n>XY, +6, +8, +13, der(2)t(1;2)(q31;q35), ins(11;9)(q23;p22p23), del(14(q23q32.3), del(16)(q11.2q13.1); sdl with der(2) but with tetrasomy 8 and/or i(8)(q10); carries insertional variant of t(9;11) recurrent in AML, effecting rearrangement of KMT2A (MLL) with MLLT3 (AF9); ins(11;9) plus trisomies 8 and 13 also present in sister cell line MOLM-13 which it closely resembles | human hyperdiploid karyotype with 4% polyploidy - 51(48-52)<2n>XY, +8, +8, +8, +13, del(8)(p1?p2?), ins(11;9)(q23;p22p23) - resembles published karyotype - sideline with idem, +19 - carries occult insertion effecting KMT2A-MLLT3 (MLL-MLLT3; MLL-AF9) fusion | human near-tetraploid karyotype - 94(88-96)<4n>XY/XXY, -Y, +1, +3, +6, +6, -8, -13, -19, -22, -22, +2mar, add(1)(p11), del(1)(q42.2), i(2q), del(6)(p21)x2-4, i(7p), der(9)t(9;11)(p22;q23)i(9)(p10)x2, der(11)t(9;11)(p22;q23)x2, add(12)(q24)x1-2, der(13)t(8;13)(p11;p12), add(?18)(q21) - carries t(9;11) associated with AML M5 | human hypertetraploid karyotype - 93(83-96)<4n>XXYY, +3, +5, +5, +6, +6, +7, -9, -10, -11, -12, +13, -14, -17, -21, +22, +22, +mar, del(1)(p11), i(5p)x2-3, der(6)t(6;15)(p25;q24), der(13)ins(13;13)q32;q?q?)x3-4 - no cytogenetic evidence of PML-RARA fusion - resembles published karyotype | human hypotriploid karyotype with sharp mode - 61-68<3n>XX, -X, -3, +7, -13, -18, +3mar, del(9)(p11/13), der(14)t(14;?)(p11;?), der(17)t(17;?)(p11/13;?), der(?18)t(15;?18)(q21;?q12), del(X)(p22) - two markers appear from FISH to have arisen from Ph; fusion gene BCR-ABL1 is co-amplified together with MIR17HG (13q31) | human hypodiploid karyotype with 4.5% polyploidy - 45(42-47)<2n>X/XY, -4, +8, +8, -12, -17, -20, +2mar, der(5;17)(q10;q10)del(5)(q?11q?13), dup(7)(q12q33), del(7)(q22q35), i(8q)x2, der(8)t(6;8)(p11;q22), der(8)t(8;12)(p11;q13), der(11)t(1;11)(q13-21;p11-p13), der(16)t(?12;16)(?p13;q13/21) - resembles published karyotype | human hyperdiploid karyotype - 48(45-50)<2n>X/XY, +1, +5, +8, der(1)t(1;18)(p11;q11), i(5p), del(13)(q13q21), dup(17)(q21q25) - sideline with r(Y)x1-2 - hemizygous for RB1 | pseudodiploid human cell line. The modal chromosome number is 46, occurring in 74% with polyploidy at 5.3%. The karyotype is 46,XY,-2,-18,del(2) (p21p23),del(18) (p11.2). Most cells had normal X and Y chromosomes. |

**Supplementary Table 1.** Characteristics of the Cell Lines Used in This Study (Species, Cell Type, Tissue of Origin, and Cytogenetic Features)

**Supplementary Figure S1**. Recovery of viable cell expansion after release from the GelMA spheroids. (**A**) Experimental workflow. K562 and PL-21 GelMA 5% spheroids were enzymatically dissociated and replated under standard 2D suspension culture conditions. Viable cell counts were performed at 0, 24 and 48h; Created in BioRender. Germain, N. (2026) https://BioRender.com/q9t2k0s (**B**) Viable cell expansion expressed as fold change relative to T0 after replating (n=3, mean +/- SD); (**C**) Absolute viable cell numbers after replating. GelMA-derived cells progressively increased in number after release from the 3D microenvironment, supporting the partial reversibility of the GelMA-induced growth-restricted phenotype (n=3, mean +/- SD two-way ANOVA, with experimental condition and time as factors, followed by Šídák’s multiple-comparisons test). This assay provides population-level evidence of recovery but does not directly assess S-phase entry at the single-cell level.
